# Brugada Syndrome in the Pediatric Population: Diagnostic Challenges and Clinical Outcome—Results of the German Multicenter Study COGIA

**DOI:** 10.1111/jce.70484

**Published:** 2026-08-30

**Authors:** Lea Lippert, Tobias Burkard, Franziska Markel, Volker Debus, Fridrike Stute, Anna Kamphues, Robert Dalla Pozza, Stefan Rupp, Maria B. Gonzalez y Gonzalez, Claudia Junge, Majed Kanaan, Kirstin Hoff, Anja Hanser, Sven Dittmann, Dominik Sebastian Westphal, Isabel Diebold, Sebastian Clauß, Stefanie Kessner, Marc‐Phillip Hitz, Kolja Becker, Stefan Kääb, Peter Ewert, Michael Hofbeck, Wolfgang Wällisch, Sven Dittrich, Anselm Uebing, Stephan Neumann, Jan‐Hendrik Nürnberg, Joachim Hebe, Rainer Kozlik‐Feldmann, Philipp Beerbaum, Gunter Kerst, Eric Schulze‐Bahr, Roman Gebauer, Gabriele Hessling, Cordula Maria Wolf

**Affiliations:** ^1^ Department of Congenital Heart Defects and Pediatric Cardiology German Heart Center Munich, TUM University Hospital, School of Medicine & Health, Technical University of Munich Munich Germany; ^2^ European Reference Network for Rare, Low Prevalence, or Complex Diseases of the Heart (ERN GUARD‐Heart) Germany; ^3^ Department of Pediatric Cardiology Heart Center Leipzig, University of Leipzig Germany; ^4^ Department of Pediatric Cardiology University Hospital Münster Münster Germany; ^5^ Clinic for Children's Heart Medicine and Adults with Congenital Heart Disease University Heart & Vascular Center Hamburg, University Hospital Hamburg‐Eppendorf Hamburg Germany; ^6^ Division of Pediatric Cardiology and Intensive Care University Hospital, LMU Munich Munich Germany; ^7^ Pediatric Heart Center, Center for Congenital Heart Disease University Hospital Giessen and Marburg, Justus‐Liebig‐University Giessen Giessen Germany; ^8^ Department of Pediatric Cardiology and Pediatric Intensive Care Medicine Hannover Medical School Hannover Germany; ^9^ Department of Pediatric Cardiology University Hospital Aachen Aachen Germany; ^10^ Institute of Human Genetics University Hospital Schleswig‐Holstein, Campus Kiel Kiel Germany; ^11^ Department of Pediatric Cardiology and Intensive Care Medicine University Hospital Tübingen Tübingen Germany; ^12^ Department of Cardiovascular Medicine Institute for Genetics of Heart Diseases, University Hospital Münster Münster Germany; ^13^ Department of Human Genetics Landeskrankenhaus University Hospital, Paracelsus Medical University Salzburg Austria; ^14^ Institute of Human Genetics, Klinikum rechts der Isar TUM University Hospital Munich, School of Medicine & Health, Technical University of Munich Munich Germany; ^15^ Department of Internal Medicine I Klinikum rechts der Isar, TUM University Hospital Munich, School of Medicine & Health, Technical University of Munich Munich Germany; ^16^ Department of Medicine I University Hospital, LMU Munich Munich Germany; ^17^ Institute of Surgical Research at the Walter‐Brendel‐Center of Experimental Medicine University Hospital, Ludwig‐ Maximilians‐University Munich Munich Germany; ^18^ German Center for Cardiovascular Research (DZHK), Partner Site Munich, Munich Heart Alliance (MHA) Munich Germany; ^19^ Interfaculty Center for Endocrine and Cardiovascular Disease Network Modelling and Clinical Transfer (ICONLMU) Ludwig Maximilians‐University Munich Munich Germany; ^20^ Real World Evidence Germany GmbH Munich Germany; ^21^ Department of Congenital Heart Disease and Pediatric Cardiology University Hospital Schleswig‐Holstein, Campus Kiel Kiel Germany; ^22^ Institute of Medical Genetics Carl von Ossietzky University Oldenburg Oldenburg Germany; ^23^ Department of Pediatric Cardiology University Hospital Erlangen, FAU‐Erlangen‐Nürnberg Erlangen Germany; ^24^ German Center for Cardiovascular Research (DZHK), Partner Site Hamburg/Lübeck/Kiel Hamburg Germany; ^25^ Department for Pediatric and Congenital Heart Electrophysiology Center for Electrophysiology at Heart Center Bremen Bremen Germany; ^26^ Congenital Heart Center Stuttgart, Olgahospital, Klinikum Stuttgart Germany

**Keywords:** Brugada syndrome, children, diagnosis, genetics, major arrhythmic events

## Abstract

**Introduction:**

Diagnosis and treatment of children and adolescents with suspected Brugada syndrome (BrS) is challenging. Aim of the study was to evaluate the applicability of current diagnostic criteria for pediatric patients with suspected BrS in the German multicenter study COGIA and to assess clinical outcome.

**Methods:**

Data analysis of 76 pediatric patients from 12 German tertiary care centers. Primary outcome was defined by the occurrence of a major arrhythmic event (MAE), secondary outcome by symptoms, pharmacotherapy and implantation of devices. MAE was defined as sudden cardiac death (SCD), aborted cardiac arrest (ACA) and appropriate implantable cardioverter‐defibrillator (ICD) therapy.

**Results:**

Criteria for BrS diagnosis were fulfilled in 39/76 pediatric patients (51.3%) according to ESC Guidelines, including 34 patients (44.7%) according to Shanghai Score. Genetic testing identified a (likely) pathogenic *SCN5A* variant in 31/66 tested children (47.0%) (14/30 with clinical diagnosis (46.7%) and 17/36 without clinical diagnosis (47.2%)). Twenty‐four patients (31.6%) showed a spontaneous type 1 ECG pattern, in 6 patients (7.9%) it was induced by fever. Ajmaline challenge was positive in 19/25 patients (76.0%). Syncope occurred in 15 patients (19.7%) and an ICD was implanted in 7 pediatric patients (9.2%). Four patients (5.3%) experienced a MAE (ACA), all of them fulfilling diagnostic criteria. “Symptoms at first consultation” was identified as an independent risk factor for MAE in the whole cohort (HR = 11.3, *p* = 0.04).

**Conclusion:**

Establishing the diagnosis of BrS remains challenging in the pediatric cohort. MAE occurred in 5.3% of patients with suspected BrS. Symptoms at first consultation increased risk for MAE.

**Clinical Trial Registration:** The study is registered at the German Clinical Trial Register (DRKS) of the Federal Institute for Drugs and Medical Devices (BfArM), DRKS‐ID DRKS00028138.

## Graphical abstract

The study examines 76 patients of the German multicenter study COGIA with suspected BrS in 12 centers in Germany. Thirty‐nine patients met the current ESC diagnostic criteria, 34 of whom met the Shanghai Score criteria. Four major arrhythmic events (ACA, SCD, appropriate ICD shock) occurred in the cohort (5.3%), all in the group of patients fulfilling current diagnostic criteria. Risk factor analysis showed an increased risk of MAEs in patients with symptoms at the initial examination. In summary, diagnosis in children and adolescents with BrS remains challenging.

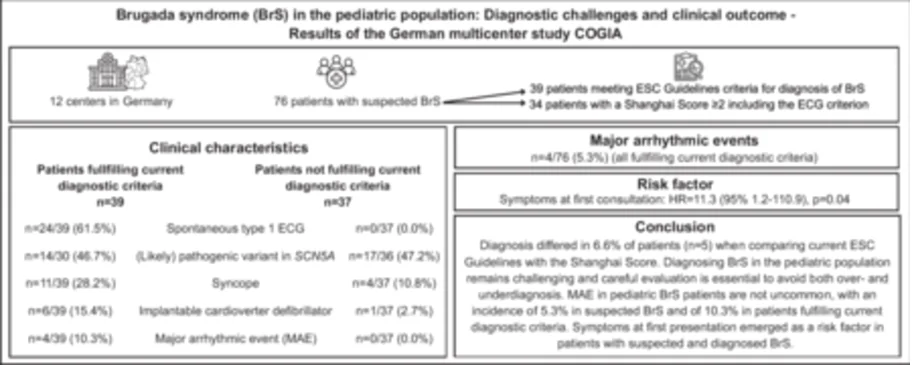

## 1 Introduction

The early and correct diagnosis of arrhythmogenic disorders is important for further clinical management and for family members, as these disorders are commonly genetically determined. Brugada syndrome (BrS) is a rare arrhythmia syndrome associated with an increased risk of sudden cardiac death (SCD) and is characterized by a diagnostic type 1 electrocardiogram (ECG) pattern [1]. In the pediatric population, the prevalence is estimated to be quite low (1: 22 000) [2]. A pathogenic loss‐of‐function variant in the cardiac sodium channel gene *SCN5A* is found in a subset of BrS patients and currently the main inherited cause; the majority of patients (> 70%), however, still remain gene‐elusive [3, 4]. The first life‐threatening arrhythmia often occurs in adulthood around the age of 38 to 48 years [5]. Nevertheless, children are also affected by this disease and may develop symptoms, especially triggered during fever [6, 7]. Diagnosing BrS at this early stage of life is challenging since the diagnostic ECG pattern might be transient and age‐dependent in disease penetrance. In contrast to congenital long QT syndrome, the diagnosis of BrS cannot be made on the presence of a (likely) pathogenic (LP/P) gene variant [8].

Currently, the diagnosis of BrS is based on two diagnostic tools—the European Society of Cardiology (ESC) Guidelines [8] and the Shanghai Score [9, 10]—but neither of these is specifically designed for children and adolescents.

This raises the question of how suitable these tools might be for the pediatric population. Furthermore, as children represent a particularly vulnerable group, more knowledge is needed about their clinical course and outcome in order to optimize risk stratification and counseling. Therefore, the current study evaluates data on children and adolescents in Germany with suspected BrS in order to gain deeper insights into these aspects and to evaluate current diagnostic standards.

## 2 Methods

### 2.1 Study Population

The national cohort study COGIA (**C**linical course, **O**utcome and **G**enetics of **I**nherited **A**rrhythmias in children and adolescents) is a retrospective data collection of pediatric patients with inherited arrhythmia syndromes. It includes patients from 12 national tertiary care centers in Germany. Inclusion criterion for this analysis was a suspected or definitive diagnosis of BrS before the age of 18 years. Patients with a relevant structural heart defect on echocardiography were excluded.

An analysis of long QT syndrome patients in the COGIA cohort study has been published previously [11].

### 2.2 Data Collection

Screening of patients at participating centers was conducted using a list of ICD‐10 codes (Version 2021, German modification) corresponding to inherited arrhythmia syndromes (see Supporting data online, Table S1) and upon local databases. Relevant information was anonymized, extracted from medical records and transferred into the electronic case report form. Compliance with the two‐person verification principle was ensured by two independent investigators (T.B. and L.L.), who reviewed eligibility criteria and confirmed entry of data relevant to the primary endpoints.

Baseline variables comprised demographic data, family history, genetic findings, indication for first presentation and timing of initial contact and diagnosis.

Follow‐up information included survival status, hospital admissions, arrhythmia‐related procedures, clinical manifestations, antiarrhythmic therapy and occurrence of arrhythmias. The last follow‐up date corresponded to the latest physician contact.

Genetic test results were classified as positive if a LP/P variant (American College of Medical Genetics and Genomics (ACMG) class 4 or 5) was identified and negative (gene‐elusive) if either a variant of uncertain significance (VUS; ACMG class 3) or no variant in the *SCN5A* gene was detected. All variants were reviewed by a clinical geneticist according to current ACMG criteria. The current classification of the variant was used for analysis [12].

The diagnosis of BrS was retrospectively evaluated according to the definition of the current ESC Guidelines [8] showing (1) a spontaneous type 1 ECG pattern at nominal or high leads or (2) an induced type 1 ECG + sudden cardiac arrest due to tachycardia (ventricular fibrillation or polymorphic ventricular tachycardia) (so‐called class I recommendations) [13] or when reaching a Shanghai Score ≥ 3.5 points (= definitive diagnosis) including the mandatory ECG criterion [9].

Additionally, patients with a Shanghai Score of 2–3 points (= probable diagnosis) and including at least one criterion in the ECG category, as well as patients who fulfilled the class IIa and IIb recommendations of the ESC Guidelines, were included and classified as affected. The class IIa recommendations (“BrS should be considered”) contain patients with an induced type 1 ECG pattern plus at least one of the following: arrhythmic syncope, nocturnal agonal respiration, family history of BrS or a family history of SCD (< 45 years old) with a negative autopsy and circumstances suspicious for BrS, whereas the class IIb recommendation (“BrS may be considered”) refers to patients who present solely with an induced type 1 ECG pattern.

### 2.3 Outcomes

The primary endpoint was the occurrence of a major arrhythmic event (MAE) before the age of 18 years, defined as SCD, aborted cardiac arrest (ACA) or appropriate shock of a implantable cardioverter‐defibrillator (ICD). Secondary endpoints as measures for morbidity included arrhythmogenic syncope, pharmacotherapy and implantation of an ICD.

If multiple ECG patterns were seen in a patient, the patient was assigned to the ECG pattern that is ranked highest in the Shanghai Score.

### 2.4 Statistics

Descriptive analyses were performed for all variables. Continuous data are presented as medians with interquartile ranges (IQR). Categorical data are summarized as absolute and relative frequencies.

To compare patients fulfilling diagnostic criteria vs. not fulfilling diagnostic criteria, sensitivity analyses were conducted. Group differences were assessed using Fisher's exact test for categorical variables and Mann–Whitney *U* test for continuous variables. Event‐free survival with respect to MAE was estimated by Kaplan–Meier analysis. The log‐rank test was not performed, as no event occurred in one group (patients not fulfilling current diagnostic criteria). A Cox proportional hazards model was constructed using a forward‐selection approach. The variables evaluated for potential inclusion in the model were syncope, a spontaneous type 1 ECG pattern, symptoms at first consultation and LP/P variant in the *SCN5A* gene. A *p*‐value of < 0.05 (two‐sided) was considered statistically significant. Missing data were not imputed. All analyses were performed using SPSS version 31.0.0 (IBM Corp., Armonk, NY, USA).

## 3 Results

### 3.1 Study Population

Seventy‐six children and adolescents (50 males (65.8%)) with initially suspected BrS were included, belonging to 60 different families (Table 1 and Figure 1). Median age at first presentation was 6.2 years (IQR 1.8–11.5 years). The majority of patients (*n* = 48; 63.2%) were investigated because of familial cascade screening due to a positive family history for BrS/SCD, 14 additional patients (18.4%) had clinical symptoms for further evaluation and 14 patients (18.4%) were examined due to incidental findings or questionable symptoms. Median follow‐up time was 3.7 years (IQR 0.9–10.6 years) and median age at last follow‐up was 13.3 years (IQR 7.9–17.0 years). The observation period ranged from 2001 to 2022.

**Table 1:** Demographic data.

			Total	Patients with suspected BrS not fulfilling current diagnostic criteria	Patients fulfilling current diagnostic criteria	*p*‐value
Patients			76	37	39	
Sex male			50 (65.8)	21 (56.8)	29 (74.4)	0.15*
Age at diagnosis/first presentation			6.2 (1.8–11.5)	4.7 (0.6–10.4)	8.2 (3.3–12.9)	0.12^+^
Age at last follow‐up			13.3 (7.9–17.0)	11.4 (7.1–14.3)	14.8 (10.1–18.8)	< 0.01^+^
Reason first encounter	Incidental findings		14 (18.4)	3 (8.1)	11 (28.2)	0.04*
	Symptoms		14 (18.4)	2 (5.4)	12 (30.8)	< 0.01*
	Cascade screening		48 (63.2)	32 (86.5)	16 (41.0)	< 0.01*
Genetic testing (*n* = 66)	(Likely) pathogenic variant (ACMG class 4/5)		31 (47.0)	17 (47.2)	14 (46.7)	1.00*
	Missense		7 (24.1)	4 (23.5)	3 (25.0)	0.67*
	Nonsense		8 (27.6)	4 (23.5)	4 (33.3)	0.41*
	Splice		3 (10.3)	0 (0.0)	3 (25.0)	0.03*
	Frameshift		11 (37.9)	9 (52.9)	2 (16.7)	0.30*
	Inframe deletion		0 (0.0)	0 (0.0)	0 (0.0)	—
	VUS (ACMG class 3)		23 (34.8)	18 (50.0)	5 (16.7)	< 0.01*
	Missense		17 (73.9)	13 (72.2)	4 (80.0)	0.37*
	Nonsense		0 (0.0)	0 (0.0)	0 (0.0)	—
	Splice		3 (13.0)	2 (11.1)	1 (20.0)	1.00*
	Frameshift		1 (4.3)	1 (5.6)	0 (0.0)	1.00*
	Inframe deletion		2 (8.7)	2 (11.1)	0 (0.0)	1.00*
	No disease‐causing genetic variant for BrS		12 (18.2)	1 (2.6)	11 (36.7)	< 0.01*
Family history	First‐ or second‐degree relative with BrS		54 (71.1)	32 (86.5)	22 (56.4)	< 0.01*
	First‐ or second‐degree relative with SCD < 45 years		4 (5.3)	1 (2.7)	3 (7.7)	0.61*
ECG finding	Spontaneous type 1 ECG		24 (31.6)	0 (0.0)	24 (61.5)	< 0.01*
	Induced type 1 ECG by fever		6 (7.9)	0 (0.0)	6 (15.4)	0.03*
	Type 2 ECG converted in type 1 by ajmaline challenge		4 (5.3)	0 (0.0)	4 (10.3)	0.12*
	No type 2 ECG before, but type 1 by ajmaline challenge		5 (6.6)	0 (0.0)	5 (12.8)	0.06*
Medication	Cardiac medication		3 (3.9)	0 (0.0)	3 (7.7)	0.24*
	Other medication		3 (3.9)	1 (2.7)	2 (5.1)	1.00*
Ajmaline challenge (*n* = 25)	Positive		19 (76.0)	0 (0.0)	19 (100.0)	< 0.01*
Syncope			15 (19.7)	4 (10.8)	11 (28.2)	0.08*
Implantable loop recorder			11 (14.5)	3 (8.1)	8 (20.5)	0.19*
ICD			7 (9.2)	1 (2.7)	6 (15.4)	0.11*
	Age at implantation		10.7 (4.0–17.0)	17.0 (17.0–17.0)	10.5 (4.0–10.7)	0.32^+^
	Reason for implantation (*n* = 7)	Primary prevention	5 (71.4)	1 (100.0)	4 (66.7)	1.00*
		Secondary prevention	2 (28.6)	0 (0.0)	2 (33.3)	1.00*
MAE			4 (5.3)	0 (0.0)	4 (10.3)	0.12*
	Age at MAE		2.5 (1.3–9.6)	—	2.5 (1.3‐9.6)	—

*Note:* Data expressed as *n* (%) or median (IQR), total number 76 unless otherwise stated; *comparison was made using Fisher's exact test; ^+^comparison was made using Mann–Whitney *U* test.

Abbreviations: ACMG, American College of Medical Genetics and Genomics; BrS, Brugada syndrome; ECG, electrocardiogram; ICD, implantable cardioverter‐defibrillator; MAE, major arrhythmic event; SCD, sudden cardiac death; VUS, variant of uncertain significance.

**Figure 1:**
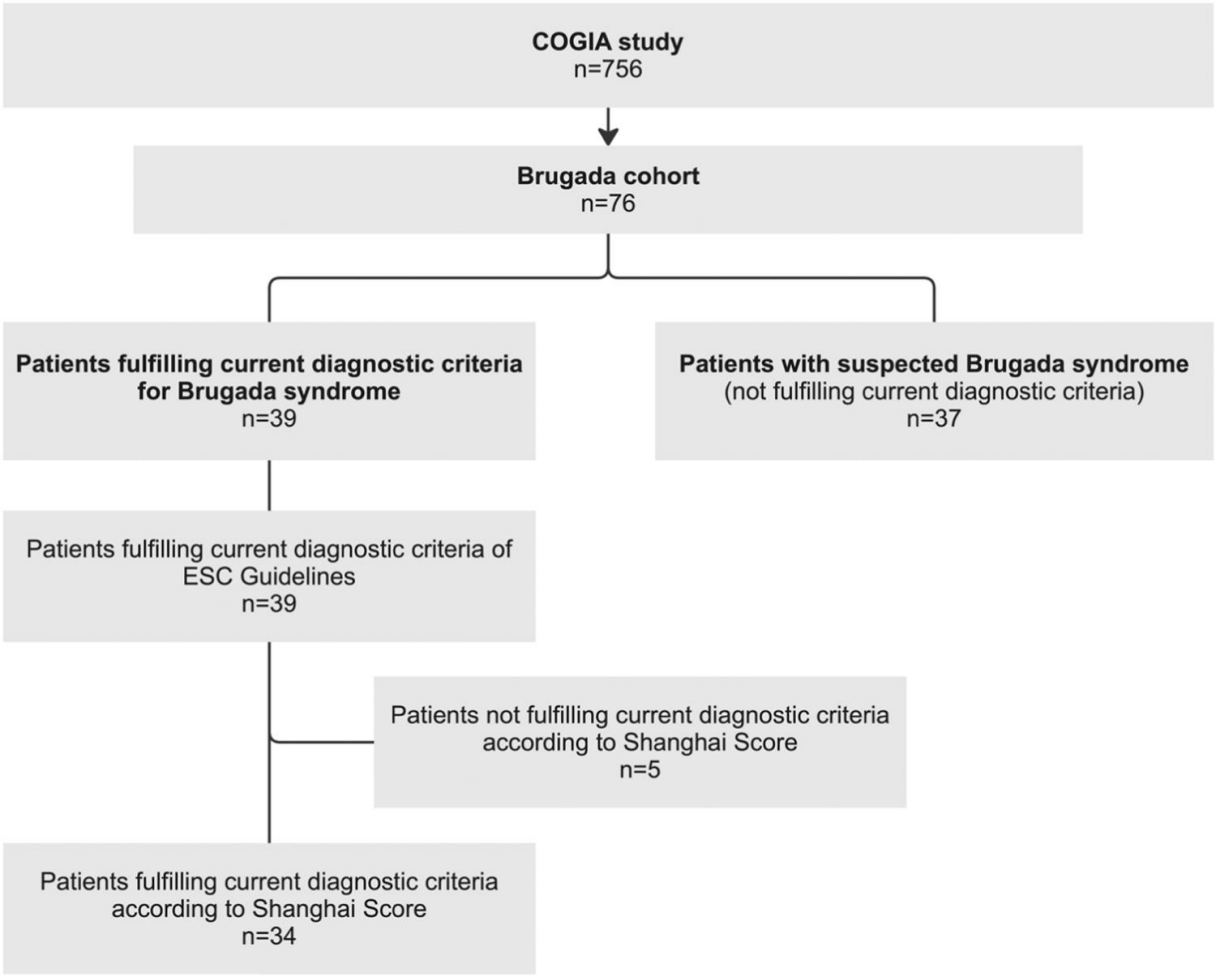
Study population. Illustrated is the study population of patients with suspected Brugada syndrome from the German multicenter study COGIA, subdivided into patients fulfilling the current diagnostic criteria and those not fulfilling current diagnostic criteria. COGIA, clinical course, outcome and genetics of inherited arrhythmias in children; ESC, European Society of Cardiology.

### 3.2 Diagnosis

Thirty‐nine patients (51.3%) fulfilled the current criteria for a BrS diagnosis according to the ESC Guidelines [8] (see Methods), and 34 patients (44.7%) according to a sufficiently high Shanghai Score (Figure 2) [9]. The five patients meeting the ESC diagnostic criteria but not the Shanghai Score criteria for a BrS diagnosis exhibited an induced type 1 ECG following ajmaline i.v., while no type 2 or type 3 ECG had been recorded before or afterwards. All BrS patients diagnosed according to the Shanghai Score also fulfilled the ESC diagnostic criteria.

**Figure 2:**
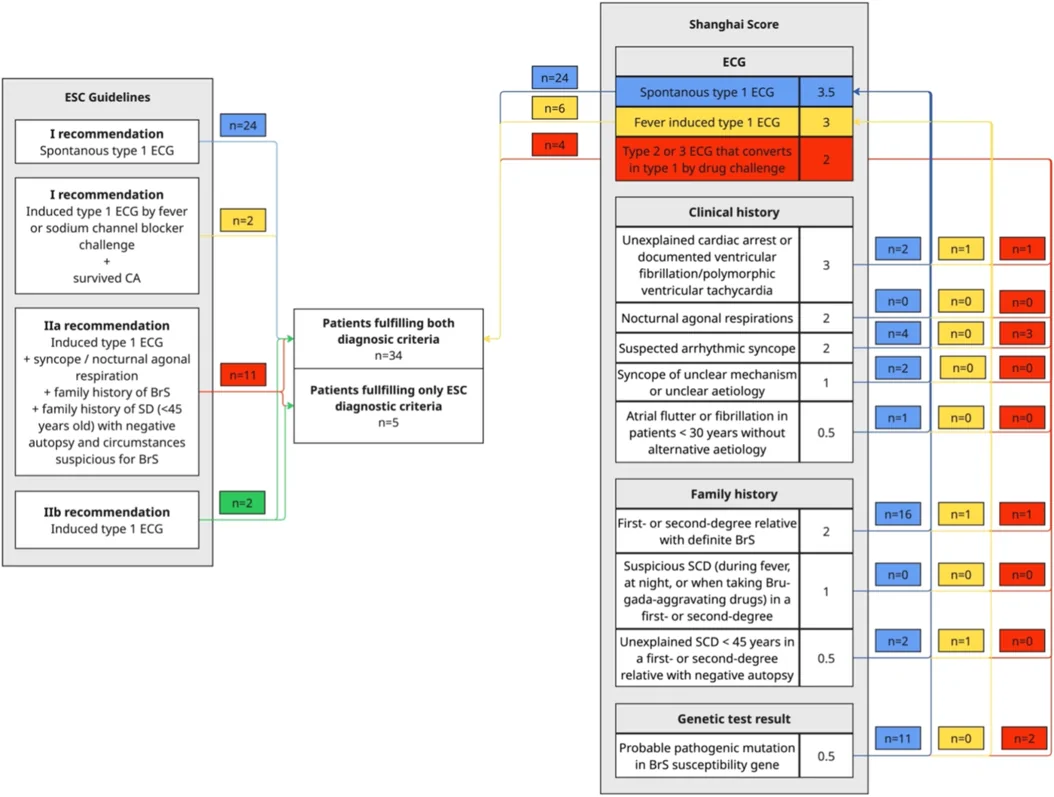
Comparison of ESC Guidelines and Shanghai Score with COGIA patient data. The figure shows the comparison of COGIA patients who fulfilled the current diagnostic criteria by the ESC Guidelines or the Shanghai Score. Several numbers for the categories right to the Shanghai Score graph may belong to one patient case. BrS, Brugada syndrome; CA, cardiac arrest; ECG, electrocardiogram; ESC, European Society of Cardiology; SCD, sudden cardiac death; SD, sudden death.

Within the total cohort of 76 pediatric or adolescent patients, 37 (48.7%) did not meet the diagnostic criteria for BrS. Both groups were further compared for their distinct features.

### 3.3 Genetic Testing

Genetic testing was performed in 66 out of 76 patients (86.8%) and was carried out from 2001 to 2021 at a median age of 5.3 years (IQR 1.5–10.7 years).

In 31 of these patients (47.0%), a LP/P *SCN5A* variant was detected. Among them, 14 patients (45.2%) fulfilled the diagnostic criteria for BrS and showed disease penetrance, whereas 17 patients (54.8%) currently did not meet these criteria and are thereby gene variant carriers for BrS. Frequency of LP/P variants detected in genetic testing was highest in patients who were tested due to incidental findings (55.6%), followed by cascade screening (48.9%) (see Supporting data online, Table S2).

The *SCN5A* gene variants displayed a highly heterogeneous pattern, and no clustering of variants within any specific gene region was noted (Figure 3).

**Figure 3:**
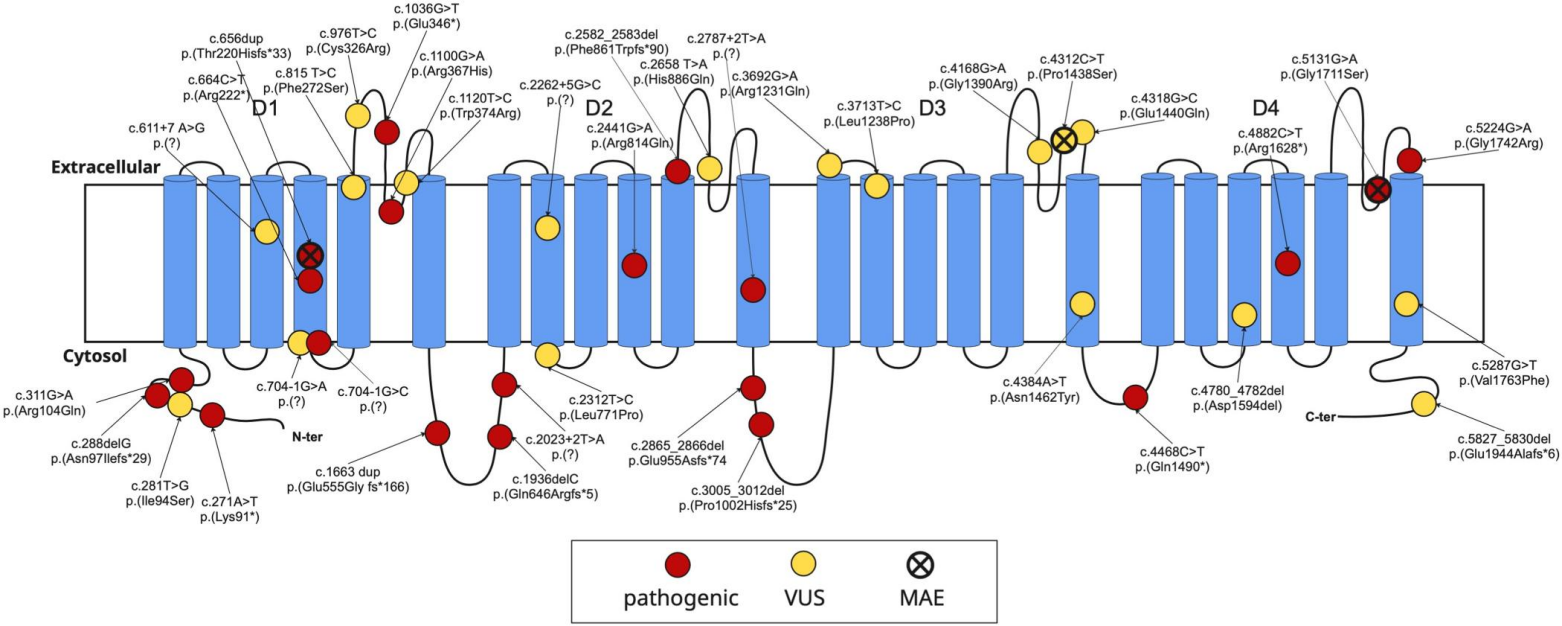
Location of (likely) pathogenic variants and VUS in *SCN5A*. Depicted are all variants in *SCN5A* found in the COGIA cohort. Each point represents a variant found in a patient. The variants c.271 A > T p.(Lys91*), c.1663 dup p.(Glu555Gly fs*166), c.2312 T > C p.(Leu771Pro), c.2582_2583del p.(Phe861Trpfs*90), c.2658 T > A p.(His886Gln), c.4384 A > T p.(Asn1462Tyr), c.4780_4782del p.(Asp1594del), c.4882 C > T p.(Arg1628*), c.5224 G > A p.(Gly1742Arg) and c.5287 G > T p.(Val1763Phe) appeared in several family members in the study. The label “pathogenic” includes ACMG class 4 (likely pathogenic) and 5 (pathogenic). All variants are annotated to the transcript NM_000335.5. The classification shown (pathogenic/VUS) refers to the current reclassified report. Truncating variants lead to a shortened protein. Consequently, when they occur in a patient, the channel is not fully formed as shown here. MAE, major arrhythmic variant; VUS, variant of uncertain significance.

Variant reclassification was performed and resulted in downgrading from LP/P to a VUS in 20 cases (26.3%) and to a benign variant in one case (1.3%) during the observation period.

### 3.4 ECG Findings

Twenty‐four patients (31.6% of the total cohort or 61.5% of the patients fulfilling current diagnostic criteria) showed a spontaneous type 1 BrS ECG (Table 1). In addition, a type 1 ECG induced by fever was seen in 6 patients (7.9%) whereas 4 patients (5.3%) showed a type 2/3 BrS ECG at baseline that converted into a type 1 ECG after ajmaline testing.

Ajmaline provocation testing was performed in 25 patients (32.9%) at a median age of 11.6 years (IQR 9.8–14.3 years). In 19 patients (76.0%) drug testing was positive by detection of a type 1 ECG. No patient experienced any complications during the procedure.

### 3.5 Outcome

Four patients (5.3% of the whole cohort) who finally all met the diagnostic BrS criteria experienced a MAE (Table 2). In contrast, none of the patients with suspected BrS who were not fulfilling diagnostic criteria, experienced a MAE during follow‐up. Consequently, MAE rate was 10.3% in 39 pediatric BrS patients, the median age was 2.5 years (IQR 1.1–12.8 years). In all patients, MAE occurred prior to the definitive diagnosis of BrS.

**Table 2:** Characteristics of patients with a MAE.

Patient	Age at dia‐gnosis	Family history	Syncope in medical history	ECG findings	Genetic testing	Age at MAE	Circumstances of MAE	Additional information
1, female	3 years	Unclear	Yes	Type 2 ECG, induced to type 1 in ajmaline challenge	*SCN5A*, NM_000335.5; c.656dup p.(Thr220Hisfs*33), heterozygous, likely pathogenic	3 years	Ventricular tachycardia during infection with fever and following resuscitation	Ajmaline challenge positive, ICD implanted after MAE; complications with ICD
2, male	1 year	Unclear	No	Type 1 ECG during anesthesia in seizure; transient spontaneous and induced by fever	Negative for BrS, positive variant for epileptic encephalopathy	1 year	Hospitalization after seizure, continuous ECG monitoring, initially with changes in the morphology of the ventricular complexes, widened, bundle branch block‐like and BrS‐like, then increasing AV conduction time to second‐degree AV block with 2:1 conduction, then third‐degree AV block and resuscitation	Implantation of a pacemaker
3, male	15 years	Negative	Yes, multiple; but could also have had alternative etiologies, e.g., orthostatic	Spontaneous type 1 ECG pattern	*SCN5A*, NM_000335.5, c.4309 C > T, p.(Pro1437Ser), heterozygous, VUS	15 years	History of multiple syncope prior to ACA, lay resuscitation and ROSC, in terms of differential diagnosis the event might also be due to sinus deceleration which he also presented after ICD implantation	Secondary prophylactic ICD implantation
4, male	11 months	Positive: Mother and brother with BrS, grandfather with SCD (age of 36 years)	No	Spontaneous type 1 ECG pattern	*SCN5A*, NM_000335.5, c.5131 G > A, p.(Gly1711Ser), heterozygous, likely pathogenic	11 months	After sudden vomiting, loss of muscle tone, consciousness and pulse, lay resuscitation, recording of ventricular fibrillation; ROSC in hospital	After ACA neurological residual syndrome with severe developmental disorder, tetraspastic cerebral palsy, and therapy‐refractory structural epilepsy

Abbreviations: ACA, aborted cardiac arrest; AV, atrioventricular; BrS, Brugada syndrome; ECG, electrocardiogram; ICD, implantable cardioverter‐defibrillator; MAE, major arrhythmic event; ROSC, return of spontaneous circulation; SCD, sudden cardiac death; TdP, torsades de pointes; VUS, variant of uncertain significance.

### 3.6 Patients Fulfilling Current Diagnostic Criteria

Thirty‐nine patients (51.3%) were fulfilling the current diagnostic criteria for BrS and were diagnosed at a median age of 8.2 years (IQR 3.3–12.9 years). Genetic testing revealed a LP/P variant in 14 patients (46.7% of 30 tested patients). Eleven patients (28.2%) had a syncope, 8 patients (20.5%) received an implantable loop recorder, and in 6 (15.4%) patients an ICD was implanted. In 4 patients (66.7%) the ICD was primary prophylactic (after syncope) and in 2 patients (33.3%) implantation was secondary prophylactic after ACA. No patient received an appropriate ICD shock and no life‐threatening arrhythmias were recorded. Two patients (33.3%) suffered from device‐related complications (*n* = 1 inadequate shock; *n* = 1 dislocation).

### 3.7 Patients With Suspected Diagnosis

Thirty‐seven patients (48.7%) did not meet the current European Guidelines for diagnosis and/or a diagnosis according to the Shanghai Score. In 17 patients (47.2%) a LP/P variant was found and in 18 patients (50.0%) a VUS was detected. Eight patients (21.6%) showed a type 2/3 ECG. An implantable loop recorder was implanted in three patients (8.1%) and one patient (2.7%) received an ICD. The indication for implantation was a syncope and signs of abnormal repolarization in the ECG.

### 3.8 Patients Not Fulfilling Current Diagnostic Criteria, but a Shanghai Score ≥ 3.5

Three patients of the group of patients without a definitive diagnosis reached a Shanghai Score of 4 to 4.5 points without meeting the ECG criterion. Therefore, they do not fulfill the criteria for a definitive diagnosis. All of these patients have a family member diagnosed with BrS and presented with syncope in medical history. One of these patients carries a likely pathogenic variant in *SCN5A*. In the other two patients, the variant in *SCN5A* was downgraded to a VUS after re‐assessing current genetic criteria. A type 1 ECG pattern was not observed in any of these patients so far and an ajmaline test was not performed in those patients. Two patients received an implantable loop recorder and in one patient an ICD was implanted after syncope.

### 3.9 Probability of Event‐Free Survival and Risk Analysis for MAE

The primary outcome differed markedly between the group of patients who met the current diagnostic criteria and those who did not, as four MAEs (4/39 (10.3%)) occurred in the group of patients who met the diagnostic criteria, while no events occurred in the group of patients who did not meet the diagnostic criteria. The probability for event‐free survival was 91.4% at the age of 18 years for the whole cohort. In the group of patients fulfilling current diagnostic criteria, probability of event‐free survival was 86.3% at the age of 18 years (Figure 4).

**Figure 4:**
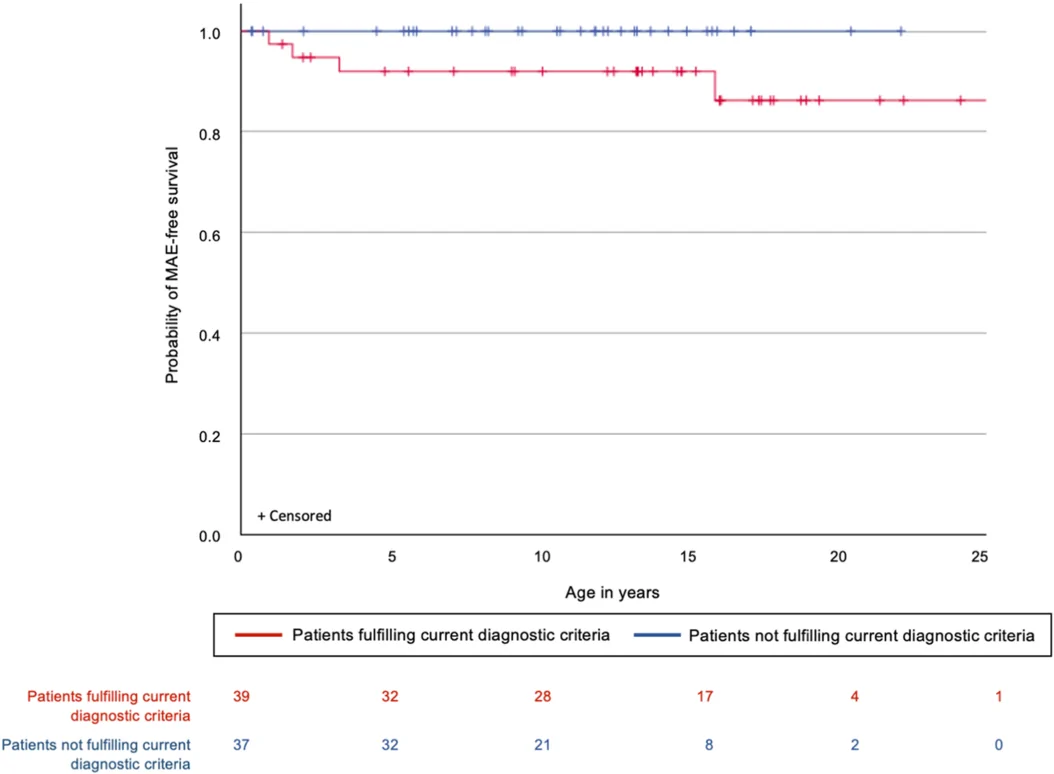
Kaplan–Meier curve for probability of MAE‐free survival. The figure shows the probability of MAE‐free survival in patients fulfilling current diagnostic criteria and not fulfilling current diagnostic criteria for BrS. MAE, major arrhythmic event.

The risk factor analysis revealed that symptomatic presentation at first consultation [HR = 11.3 (95% 1.2–110.9), *p* = 0.04] was an independent risk factor for a MAE in the entire cohort (see Supporting data online, Table S3).

Figure 5 shows a conceptual diagram integrating diagnostic and management processes.

**Figure 5:**
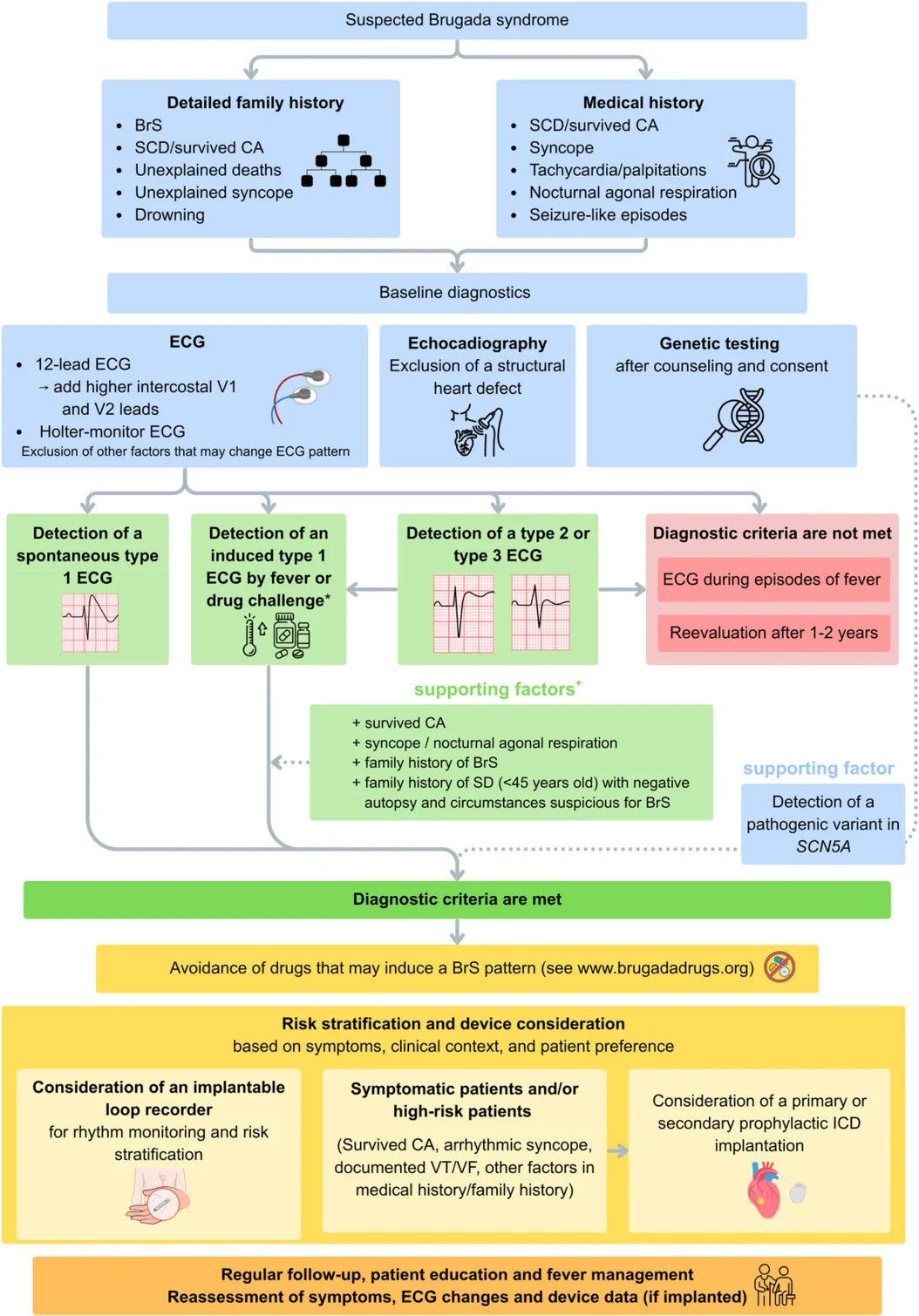
Diagnosis and management of patients with suspected Brugada syndrome. The diagram provides recommendations for the diagnosis and management of children and adolescents with suspected Brugada syndrome; *if available, consideration based on age and symptoms; ^+^use Shanghai Score or ESC Guidelines. BrS, Brugada syndrome; CA, cardiac arrest; SCD, sudden cardiac death; ECG, electrocardiogram; SD, sudden death; VT, ventricular tachycardia; ICD, implantable cardioverter‐defibrillator; VF, ventricular fibrillation.

## 4 Discussion

This study represents the largest German national cohort of children and adolescents with suspected BrS. The aim of this study was to systematically evaluate the current diagnostic standards [8, 9] in children and adolescents suspected to have BrS, as those criteria are not specifically tailored to the pediatric population. Childhood and adolescence precede the peak risk for life‐threatening events and show highly variable presentations, from asymptomatic to ACA. The study additionally reports clinical outcomes, enabling comparison with pediatric data and improving the understanding of disease course, genetics and risk for MAEs.

### 4.1 Genetics

The majority of patients underwent genetic testing as recommended [8, 13].

Comparable to published data [14], nearly two‐third of patients (63.2%) were examined due to familial cascade screening.

Genetic testing showed a similarly high yield of LP/P variants in *SCN5A* in patients who fulfilled the current diagnostic criteria and in those who did not. The yield in diagnosed patients (46.7%) was slightly lower, yet comparable to other pediatric cohorts [6, 15, 16] and, as previously reported, significantly higher than in studies involving adults [15, 17].

In a substantial number of patients (*n* = 20, 30.3%), the classification of the genetic variant changed over the observation period, primarily from pathogenic to VUS. This shows that treating physicians should re‐examine and evaluate the detected genetic variant in order to promote individualized, patient‐tailored counseling and review the diagnosis at regular intervals. It is especially relevant given that two prior studies [15, 18] identified pathogenic *SCN5A* variants as a risk factor for a MAE in children and adolescents, although our risk factor analysis found no evidence of an elevated MAE risk among carriers in our cohort.

Within *SCN5A*, no regional clustering of variants could be identified and consequently, no additional insights relevant to genetic and subsequent clinical counselling could be derived from these findings.

### 4.2 Outcomes

Overall, the outcome of the COGIA patients is in line with previously published studies [6, 14, 15, 16, 18]. The COGIA study revealed a not insignificant number of MAEs (10.3%) in patients with BrS and a rather high number of patients with syncope in medical history. It is striking that none of the patients with ICD received an adequate shock, but two had complications, including inappropriate shocks. This confirms previously published studies that reported a high rate of complications, which, as in our case, led to lead replacements or inappropriate shocks [19, 20, 21]. It once again highlights the difficulty and tension between overtreatment, which can also lead to complications, and a lack of protection.

Three MAEs occurred very early in life and in two without prior symptoms such as syncope. All four patients did not reach the diagnostic criteria before their ACA and showed their ECG findings only afterwards. Therefore, it must be acknowledged that risk stratification and modeling cannot guarantee the prevention of all adverse outcomes, especially if the life‐threatening event precedes the diagnosis.

### 4.3 Risk Factor Analysis

The probability of MAE‐free survival up to the age of 18 years was relatively high (whole cohort: 91.4%; diagnosed patients: 86.3%). In addition, there was not a single death in the data we examined of the last 20+ years. “Symptoms at first consultation” was found to be a significant risk factor in our suspected pediatric BrS patients, as it was also the case in the publication of Andorin et al. for diagnosed pediatric BrS patients [14]. This underscores the need for caution, especially in symptomatic patients, but also results from the fact that in all patients the symptoms at diagnosis were already the MAE.

Whether a spontaneous type 1 ECG in children contributes to an increased risk is assessed differently in the published studies [14, 18]. Our data cannot support the hypothesis that a spontaneous type 1 ECG is a risk factor, as no child with MAE in our cohort was reported to have had a spontaneous type 1 ECG before the MAE. Three MAE patients showed a spontaneous type 1 ECG in follow‐up. As mentioned above, carrying a LP/P *SCN5A* variant was also not found to be a risk factor in contrast to findings from other pediatric cohorts [15, 18].

No risk factors were identified if analyzing only the sub‐cohort of children fulfilling current diagnostic criteria, probably due to the small sample size with fewer MAEs than in comparable publications [14, 15, 18], which poses the major limitation of our risk factor analysis.

### 4.4 Diagnosis

This publication primarily considered the diagnostic options from the current ESC Guidelines and the use of the Shanghai Score. According to these two systems, a significant number of children (*n* = 5, 6.6%) would receive two different results in the question of definitive diagnosis or not. Whether or not there is a clear diagnosis of BrS is critical for the clinical management of the patient and for family screening.

Interpretation of ECG findings is challenging in the pediatric population as incomplete right bundle branch block can be physiologic (occurring in approximately 3% of children) resulting in a suspicion of BrS that may subsequently not be confirmed [22].

We identified patients who had a high Shanghai Score of 4 to 4.5 points for definitive diagnosis, as they had a family member with BrS and syncope, but did not meet the criteria in the ECG category. This reflects that the optimal conditions or timing for obtaining a type 1 ECG might have never been met and/or the fact that they did not undergo an ajmaline challenge. The reported tolerability and safety of ajmaline challenge in pediatric populations differ between studies so that it remains a controversial diagnostic tool in children and adolescents [23, 24, 25, 26]. An ajmaline challenge was performed in 25 of our patients (32.9%) and deemed positive in 19 patients (76.0%). There were no complications reported in our cohort. However, the interpretation of a negative ajmaline challenge in our cohort is questionable, as the ajmaline challenge can be negative in children, especially in childhood, and is only reliable towards the end of puberty and the beginning of adulthood [27]. In the ESC Guidelines, a sodium channel blocker challenge is recommended for patients with a family history of BrS or sudden arrhythmic death syndrome and for patients with resuscitated cardiac arrest without structural heart disease [8]. Current recommendation especially for children and adolescents is to test only symptomatic children with ajmaline [28].

The focus therefore remains on performing an ECG on these children in the event of fever, in order to identify a type 1 ECG, as fever is the most relevant trigger for inducing the typical ECG pattern [7, 16]. This must also be communicated to parents during consultations. Alternatively, in future, it could be discussed that a child with these clear risk factors and indicators for BrS should meet the prescribed diagnostic criteria, although the right timing for unmasking a type 1 ECG was not met so far. Yet, it is important to ensure that this does not result in overdiagnosis.

### 4.5 Limitation

All limitations of a retrospective data analysis apply to our study. In addition, although multicenter and national in study design, COGIA represents data from tertiary care centers specialized in pediatric cardiology and referral bias might influence the reported clinical outcome of patients. The risk factor analysis was complicated by the small size of the cohort and the low number of MAEs and could be repeated in a larger sample. Last, there has been changes in diagnostic possibilities, clinical management and interpretation of genetic findings over the past decades, including increasingly detailed recommendations in international guidelines. However, COGIA mainly represents young patients followed in the past two decades.

## 5 Conclusion

In a notable proportion of pediatric patients (*n* = 5, 6.6%), the diagnostic classification for BrS varied depending on the criteria applied. Data also suggest that some patients with BrS may not meet ECG‐based criteria simply because a type 1 ECG pattern was not captured at the right moment. MAEs occurred in 5.3% of the whole cohort (10.3% in the group of patients fulfilling current diagnostic criteria). Symptomatic presentation at first consultation was an independent risk factor for MAE. Overall, diagnosis in pediatric patients remains challenging—both to reliably identify patients at risk and to avoid overdiagnosis.

## Ethics Statement

The study protocol received approval from the ethics committee of the Technical University of Munich (547/21 S‐SR, approval date: September 14, 2021) as well as from the respective local ethics committees of the participating centers. Owing to the retrospective nature of the study and the use of anonymized data, the requirement for written informed consent was waived. The project was conducted in accordance with the principles of the Declaration of Helsinki and adhered to standards of good scientific practice.

## Consent

Informed consent was waived for this retrospective anonymous data collection.

## Conflicts of Interest

C.M.W. is consultant for Bristol‐Myers Squibb, Rocket Pharmaceuticals, Day One Biopharmaceuticals Inc., BioMarin Pharmaceuticals, Anacardio, Novo Nordisk, Nuevocor and Cytokinetics and has ownership interest in Preventage Therapeutics. She owns stocks in Alnylam, Idorsia, Immatics, Passage Bio and Viatris. She gets support for the present manuscript (e.g., funding, provision of study material, medical writing, article processing charges, etc.) from the research funding award 2020 awarded to C.M.W. L.L. gets support for the present manuscript (e.g., funding, provision of study material, medical writing, article processing charges, etc.) from the research funding award 2020 for C.M.W. S.K. gets support for the present manuscript (e.g., funding, provision of study material, medical writing, article processing charges, etc.) from the DZHK (German Center for Cardiovascular Research), Partner Site Munich, Munich Heart Alliance (MHA), 80802 Munich, Germany. A.U. holds leadership or fiduciary role in other board, society, committee or advocacy group, paid or unpaid in the German Society for Paediatric Cardiology. M.‐P.H. holds grants or contracts from any entity: DFG and BMBF. S.D. participates on a Data Safety Monitoring Board or Advisory Board: Hyperox, EudraCT 2021‐002071‐91. D.S.W. received honoraria for presentation of Kardioimpuls GbR, AstraZeneca GmbH, medpoint Medizinkommunikation GmbH, Boehringer Ingelheim Pharma GmbH and Bristol‐Myers Squibb GmbH.A.H. received grants from the German Herzstiftung e.V., Gerd Killian Award, TÜFF Habilitation Grant of the Faculty of Medicine, University of Tübingen and financial support from the Stiftung zur Förderung der Erforschung von Zivilisationserkrankungen.

## Supporting information


Supporting File

## Data Availability

Data cannot be made available unless each individual participating site is contacted and agrees to data sharing.
