# Supplementary material for: Brugada Syndrome in the Pediatric Population: Diagnostic Challenges and Clinical Outcome—Results of the German Multicenter Study COGIA
Source: J Cardiovasc Electrophysiol. 2026 Aug 30;37(11):2638–50. doi: 10.1111/jce.70484 (PMC13768784; doi:10.1111/jce.70484)
Supplement: Supplementary file 1 [file JCE-37-2638-s001.docx]

Supplementary material online

## Table S1

| ICD-10-CM Diagnosis Code | Description |
| --- | --- |
| I44-I45 | Sudden death with conduction disorder |
| I45.8 | Other specified conduction disorders |
| I46 | Cardiac arrest |
| I46.0 | Cardiac arrest with successful resuscitation |
| I46.1 | Sudden cardiac death, so described |
| I46.9 | Cardiac arrest, cause unspecified |
| I47 | Paroxysmal tachycardia |
| I47.2 | Ventricular tachycardia |
| I49 | Other cardiac arrhythmias |
| I49.0 | Ventricular fibrillation and flutter |
| I49.8 | Other specified cardiac arrhythmias  Brugada syndrome  Long QT syndrome |
| G72.3 | Periodic paralysis |
| R94.3 | Abnormal results of cardiovascular function studies  Prolongation of QT interval |

Title: List with ICD-10 codes (modified for Germany) for arrhythmia syndromes

Table S2

|  | Total | Incidental findings | Symptoms | Cascade screening |
| --- | --- | --- | --- | --- |
| Patients | 66 | 9 | 12 | 45 |
| (Likely) pathogenic variant | 31 (47.0) | 5 (55.6) | 4 (33.3) | 22 (48.9) |
| VUS | 23 (34.8) | 1 (11.1) | 3 (25.0) | 19 (42.2) |
| negative | 12 (18.2) | 3 (33.3) | 5 (41.7) | 4 (8.9) |

Title: Genetic variants detected according to reason for first presentation

Legend: Data expressed as n (%)

Abbreviations: VUS: variant of uncertain significance

Table S3

|  | Univariate analysis | | |
| --- | --- | --- | --- |
|  | HR | 95% CI | p-value |
| Symptoms at first consultation | 11.3 | 1.2-110.9 | 0.04 |
| Syncope | 2.9 | 0.4-21.7 | 0.29 |
| (Likely) pathogenic variant in *SCN5A* | 1.2 | 0.2-8.3 | 0.88 |
| Spontaneous type 1 ECG pattern | 1.8 | 0.3-13.0 | 0.56 |

Title: Risk factor analysis

Abbreviations: CI: confidence interval; ECG: electrocardiogram; HR: hazard ratio
